# Treg in inborn errors of immunity: gaps, knowns and future perspectives

**DOI:** 10.3389/fimmu.2023.1278759

**Published:** 2024-01-08

**Authors:** Rebeca Kennedy-Batalla, Daniel Acevedo, Yiyi Luo, Ana Esteve-Solé, Alexandru Vlagea, Rafael Correa-Rocha, Ma Elena Seoane-Reula, Laia Alsina

**Affiliations:** ^1^ Laboratory of Immune-Regulation, Gregorio Marañón Health Research Institute (IISGM), Madrid, Spain; ^2^ Clinical Immunology and Primary Immunodeficiencies Unit, Allergy and Clinical Immunology Department, Hospital Sant Joan de Déu, Barcelona, Spain; ^3^ Clinical Immunology Unit, Hospital Sant Joan de Déu-Hospital Clínic, Barcelona, Spain; ^4^ Study Group for Immune Dysfunction Diseases in Children (GEMDIP), Institut de Recerca Sant Joan de Déu, Barcelona, Spain; ^5^ Immunology Department, Biomedic Diagnostic Center (CDB), Hospital Clínic of Barcelona, Clinical Immunology Unit Hospital Sant Joan de Déu-Hospital Clínic de Barcelona, Barcelona, Spain; ^6^ Pediatric Immuno-Allergy Unit, Allergy Department, Hospital General Universitario Gregorio Marañón, Madrid, Spain; ^7^ Primary Immunodeficiencies Unit, Hospital General Universitario Gregorio Marañón, Madrid, Spain; ^8^ Department of Surgery and Surgical Specializations, Facultat de Medicina i Ciències de la Salut, Universitat de Barcelona, Barcelona, Spain

**Keywords:** Treg, IPEX syndrome, immune tolerance, primary immunodeficiency, primary immune regulatory disorders, cell-based therapies, immune dysregulation, Helios

## Abstract

Regulatory T cells (Treg) are essential for immune balance, preventing overreactive responses and autoimmunity. Although traditionally characterized as CD4+CD25+CD127^low^FoxP3^hi^, recent research has revealed diverse Treg subsets such as Tr1, Tr1-like, and CD8 Treg. Treg dysfunction leads to severe autoimmune diseases and immune-mediated inflammatory disorders. Inborn errors of immunity (IEI) are a group of disorders that affect correct functioning of the immune system. IEI include Tregopathies caused by genetic mutations affecting Treg development or function. In addition, Treg dysfunction is also observed in other IEIs, whose underlying mechanisms are largely unknown, thus requiring further research. This review provides a comprehensive overview and discussion of Treg in IEI focused on: A) advances and controversies in the evaluation of Treg extended subphenotypes and function; B) current knowledge and gaps in Treg disturbances in Tregopathies and other IEI including Treg subpopulation changes, genotype-phenotype correlation, Treg changes with disease activity, and available therapies, and C) the potential of Treg cell-based therapies for IEI with immune dysregulation. The aim is to improve both the diagnostic and the therapeutic approaches to IEI when there is involvement of Treg. We performed a non-systematic targeted literature review with a knowledgeable selection of current, high-quality original and review articles on Treg and IEI available since 2003 (with 58% of the articles within the last 6 years) in the PubMed database.

## 1 Introduction

Regulatory T cells (Treg) are a subtype of T lymphocytes essential for immune homeostasis. Due to their regulatory function, Treg are able to supress and control immune responses to restore the immune system’s steady state, and they are necessary to tolerate autoantigens. Treg are essential in physiological processes such as pregnancy, host-commensal interactions, tolerance to allergens, and tissue repair, as they help decide between what should be considered threatening or harmless and, hence, allow the immune system to mount a response or suppress it, respectively. Because they are so essential for correct immune function, Treg are also involved in numerous diseases (1, 2). Defective Treg can disturb immune balance, leading to pathologies such as allergies, autoimmune and inflammatory disorders, infections, cancer, and primary immune regulatory disorders (PIRD) (2). This review aims at providing an integrated view of Treg in the context of inborn errors of immunity (IEIs) highlighting what is known and what is not known, the problems in Treg characterisation, and how they affect understanding of underlying pathological mechanisms of IEIs. In addition, it is intended to provide insight into potential Treg cell-based treatments that could be of use in IEI management. We performed a non-systematic targeted literature review with a knowledgeable selection of current, high-quality original and review articles on Treg and IEI available since 2003 (with 58% of the articles within the last 6 years) in the PubMed database.

### 1.1 History of Treg: from CD4+ Treg to CD8+ Treg

Discovered in 1969 as thymic-derived T cells with suppressive capacity in mice (3–5), Treg have gained great interest in immune regulation and their implications in different diseases. Shortly after their discovery, during the 1970s and 1980s, it was seen that these suppressive T cells were able to slow down autoimmune disease in rodent models (6, 7). During the decade of the 1990s, several discoveries were made. First and foremost, these cells were seen to express high levels of the IL-2 receptor alpha chain (IL-2Rα, CD25) (4, 6, 8) that is now one of the markers used for Treg characterisation. Although discovery of the suppressive cytokines IL-10 and transforming factor-beta (TGF-β) was made in the 1990s, it was not until later that they were associated with Treg function (7). Despite the growing knowledge, human Treg were not discovered until 2001 both in thymus and periphery (4, 6, 7). The identification of the autoimmune scurfy mice and immune dysregulation polyendocrinopathy enteropathy X-linked (IPEX) syndrome in humans caused by loss of function of *FOXP3* pointed towards the role of this gene in Treg function (2, 6). A few years later, in 2003, FoxP3 expression was detected in Treg, in mice (6, 7) and later in humans (9, 10). FoxP3 expression was correlated with CD25 levels (7) and in 2006, thanks to the discovery of an inverse correlation between CD127 (IL-7 receptor) and FoxP3, Treg definition by surface markers was defined as follows: CD3+CD4+CD25^hi^CD127^low^ which is still the most common phenotype used for their characterization by flow cytometry (4, 8, 11). The inducible T-cell costimulator (ICOS) surface molecule was known to be expressed in activated T cells. In 2008, ICOS expression was observed in Treg. According to its expression, Treg could be divided into two subsets depending on the suppressive mechanisms used: ICOS+, which use IL-10 to suppress dendritic cells (DCs) and TGF-β to suppress T cell function, and ICOS-, which only use TGF-β (12, 13). With all the developments in Treg characterization, function, and their disease-related role, in 2013 Abbas et al. proposed, in addition to the phenotypic classification, a unified nomenclature for Treg in order to facilitate their description, dividing them depending on where they completed their development (14). According to the new classification, Treg can be thymus-derived or natural Treg (tTreg or nTreg) if they complete their development in the thymus, or peripherally derived Treg (pTreg) if development occurs in the periphery. Moreover, they can also be induced *in vitro* and called induced Treg (iTreg) (5–7, 14). tTreg comprise around 80% of circulating Treg and have stable FoxP3 expression (15). pTreg, on the other hand, express ectopic FoxP3 which is not stable and only provides suppressive functions while it is expressed (16).

In mice, FoxP3 seems to be a Treg exclusive marker which makes it possible to characterise Treg as CD25+FoxP3+. On the other hand, human conventional T cells (Tconv) can also gain FoxP3 expression and, currently, there are no distinct markers available to distinguish between true tTreg and pTreg. Classical Treg are commonly described as CD3+CD4+CD25^hi^CD127^low^FoxP3+ (4, 7, 8). However, the best way to identify these cells is by measuring the level of methylation in the Treg-specific demethylated region (TSDR) of the *FOXP3* gene. FoxP3 is not the only gene that needs to be demethylated to ensure Treg function. CTLA-4 (cytotoxic T-lymphocyte antigen 4), Eos, GITR (glucocorticoid-induced TNFR-related), and CD25 are also responsible for correct Treg function – all of which can be used as markers to define Treg by flow cytometry (16).

As previously noted, there is still a lack of specific markers to differentiate tTreg and pTreg. Recent investigations have proposed Helios as a possible differentiating factor for tTreg. Helios expression presents a high correlation with tTreg whereas pTreg are mostly Helios–. This has been further confirmed by higher complete demethylation of TSDR of the *FOXP3* gene in tTreg compared to not more than 50% in pTreg (17). However, Helios is not yet established as a definitive marker to differentiate tTreg and pTreg as many questions are still unanswered (18).

**Figure 1:**
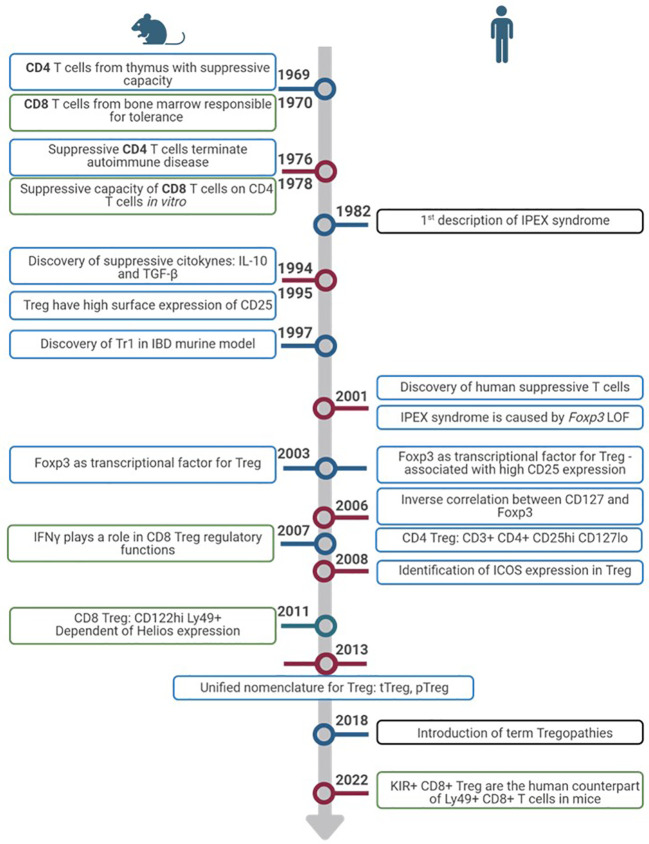
Timeline of Treg discovery. Timeline progression highlights pivotal advances in the exploration of Treg from inception to the present. Discoveries in murine models (left) and humans (right). Colors of the rectangles show advances in CD4+ Treg (blue), CD8+ Treg (green) and IEIs (black).

Although Treg research has always been more focused on CD4 T cell population, CD8 Treg populations were described in mice as early as 1970, when Gershon and Kondo described CD8 T cells from bone marrow responsible for tolerance (19, 20). In 1978, they saw CD8 suppressive capacity on CD4 cells *in vitro*, further confirmed in 1992 in an allergic encephalomyelitis animal model (21). In 2007, CD8 Treg were seen under different infections and tumour environments in rat models, and it was clear that IFNγ was important for the regulatory function of these cells (22). During this decade, it was also observed that most CD8 T cells with suppressive function were memory cells expressing high CD122 and Ly49 and that they were dependant on Helios expression, in contrast to CD4 Treg that are dependent on FoxP3 (21). These CD122^hi^ Ly49+ CD8+ T cells have been considered the “CD8 Treg” in mice. Whether this subtype exists in humans has been, and probably still is, debatable. Very recently, killer cell immunoglobulin-like receptors (KIR)+ CD8+ T cells were described as the counterpart of Ly49+CD8+ T cells in humans. Because from an evolutionary point of view KIR are the human equivalent of Ly49 in mice, these cells were studied in autoimmune and infectious human diseases such as celiac disease and COVID-19 (23). Because of the novelty of this regulatory subtype, the definition of CD8 Treg is even less established than that for CD4 Treg (24). Other human studies define CD8 Treg depending on their CD28, FoxP3, CD122, or Helios expression (24, 25). The timeline of CD4 and CD8 Treg description in mice and humans is summarized in **Figure 1** and the phenotypic markers in humans in **Table 1**.

**Table 1:** Controversies and considerations in the study of Treg and in Treg cell-based therapies in humans.

Treg phenotypic studies			
**Cell type**	**Staining**	**Different definitions**	**Reference**
CD4 Treg	SP IC IC SP	CD3+CD4+CD25hiCD127low CD3+CD4+CD25hiFoxP3+ CD3+CD4+CD25hiFoxP3+CD127low CD25hiCD127lowCCR4+	(26) (27) (28) (29)
CD4 tTreg	IC SP	CD3+CD4+CD25hiCD127lowFoxP3+Helios+ CD3+CD4+CD25hiCD127lowTIGIT+GPA33+	(30) NFS
CD4 pTreg	IC SP	CD3+CD4+CD25hiCD127lowFoxP3+Helios- CD3+CD4+CD25hiCD127lowTIGIT+GPA33-	(30) NFS
CD8 Treg	SP SP SP SP SP	CD3+CD8+CD103+ CD3+CD8+CD122+PD-1+ CD3+CD8+CD28-CD56+ CD3+CD8αα+TCRαβ+HLA-E+ CD3+CD8+KIR+	(31) (32, 33) (28, 34) (35) (23)
Treg functional studies			
**Functional effect**		**Considerations**	
T-cell proliferation		Sufficient number of cells Need to isolate cells Dose-dependent study Ensuring cell viability (susceptibility to cell death) Minimum incubation time of 3-5 days Use of CFSE as a replacement for 3H-tritylated Use of the division index as an analytical parameter	(36)
Expression of T cell activation markers		Sufficient number of cells Need to isolate cells Dose-dependent study Minimum incubation time 5-7 hours Use of activation markers like CD69 and CD154	(36)
Measurement of analytes		IFNγ and IL-2 mRNA expression Cytokine analysis in supernatant by ELISA or Luminex technology Measurement of ATP/ADP ratio	NFS
Dendritic cell activation		NFS	NFS
IL-9 signaling via the IL-9Rα receptor (STAT3 and STAT5)		NFS	NFS
Treg cell-based therapies			
**General considerations**		**Risk (R) and potential solutions (S)**	
Live cells		R: Potential instability of Treg – acquisition of T-bet, RORγt or GATA-3 S: NFS – DNA methyltransferase, IL-6, suicide casquets, integration sites.	(37, 38)
**Specific considerations**		**Risk (R) and Potential solutions (S)**	
FoxP3 destabilization		R: Acquisition of an inflammatory phenotype by Treg S: Addition of DNA methyltransferase	(37, 38)
Oncogene activation		R: Increased risk of cancer development S: NFS – Strategies to eliminate Treg in vivo if needed: insertion of suicide casquets or integration sites	(37, 38)

NFS, need further studies; IC, intracellular; SP, superficial.

### 1.2 Suppressive functions of CD4 and CD8 Treg

*FOXP3* is essential for CD4 Treg suppressive function although it is not the only gene necessary. In this context, FoxP3 expression supresses IL-2 production as well as upregulating CD25 and CTLA-4 expression – all involved in Treg mechanisms of action (16). Treg can exert their function directly or indirectly. As direct mechanisms, Tregs secrete suppressive cytokines such as IL-10, TGF-β, and IL-35 that act directly on effector T cells (Teff). They also produce perforin and granzyme-inducing apoptosis in target cells (39). Besides these secreted proteins, Tregs express a number of surface molecules that enable them to perform their inhibitory functions: through CTLA-4 and lymphocyte activation gene 3 (LAG-3) they can inhibit dendritic cells (DCs) (5) and through programmed cell death 1 (PD-1) they directly inhibit B cells (39). Moreover, the high levels of CD25 allow Tregs to highjack IL-2 preventing it from stimulating T and natural killer (NK) cells (2). As indirect mechanisms, Tregs also present ectoenzymes on their membrane, specifically CD39 and CD73, that are responsible for ATP metabolism and control Teff activation. ATP acts as an inflammatory molecule when it is cleaved, AMP acts as an anti-inflammatory signal (5, 39).

Amongst other genes necessary for Treg functionality, there is a group of molecules that enhance Treg inhibitory functions without being selective of Treg. These include ICOS, CTLA-4, PD-1, TIGIT, Helios, and LAG-3.

ICOS increases Treg effectivity and, under certain circumstances, can supress Treg apoptosis, playing an important role in their survival (7). In order to do so, ICOS needs to bind to its ligand, ICOS-L, expressed on antigen-presenting cells (APCs): DCs, B cells, and macrophages. Upon ICOS-L binding, it enhances FoxP3 transcription which, in turn, increases IL-4, TGF-β, and IL-10 secretion. ICOS+ Treg normally co-express co-inhibitory surface molecules important for their immune regulation such as CTLA-4, LAG3, PD-1, and T cell immunoglobulin and ITIM domain (TIGIT). Whether it is a matter of ICOS directly affecting this expression or indirectly contributing to the increased Treg functionality remains unclear (13).

CTLA-4 is a co-inhibitory surface molecule that is constitutively expressed in Treg. It is not a Treg-exclusive marker as it is also present in activated lymphocytes. Binding of CTLA-4 from activated lymphocytes to CD80 and CD86 present on the surface of DCs inhibits them, restoring immune homeostasis once they have completed their task. In this context, CTLA-4 acts as an immune checkpoint in order to prevent an overreactive immune system. However, the role of CTLA-4 in Treg is not completely straight forward. It is suggested that CTLA-4 from Treg binds to CD80/CD86 in DCs and downregulates their expression. Because this signal from DCs is necessary to complete T cell activation during antigen presentation, this downregulation would reduce T cell activation which, in turn, would favour regaining immune homeostasis (40).

PD-1 and its ligand PD-L1 are both highly expressed in Treg surface (7). Binding of PD-L1, on Treg surface, to PD-1, expressed on activated cells, triggers an inhibitory response that negatively affects T cell proliferation and cytotoxicity. This response induces a state of anergy on the activated T cells and can promote the induction of pTreg, by enhancing FoxP3 expression and Treg suppressive activity. Moreover, interaction of PD-1 and DCs’ PD-L1 also prompts pTreg generation (41). In turn, once pTreg are generated, their PD-1 binding to PD-L1 in DCs also generates tolerogenic DCs (7). Hence, PD-1 signalling is needed to directly and indirectly maintain immune homeostasis. PD-1/PD-L1 signalling mechanism is not exclusive to Treg, but, along with CTLA-4 and LAG-3, constitutes one of their cell-to-cell contact suppressive mechanisms (41). Constitutive PD-1 expression is related to T cell exhaustion and is usually used as a CD4+ and CD8+ T cell exhaustion marker (42).

TIGIT is another co-inhibitory receptor that is expressed in Treg but also in activated and memory T cells, NK cells, and some follicular helper T cells (Tfh). It has two ligands, CD155 (or PVR) and CD112 (or nicotine-2), although it binds with greater affinity to CD155. These molecules are expressed in APCs and T cells, among other non-hematopoietic cells (43). In the context of Treg, the effect of TIGIT binding to DCs prompts tolerogenic DCs which express IL-10 and, in turn, suppress T cell activation. Moreover, increased TIGIT expression induces *FOXP3* demethylation in Treg. It can also supress T cell priming and block the CD226 activation pathway. Without the presence of TIGIT expression, CD226 acts as an activating receptor when binding to CD155. All of these mechanisms lead to reduced T cell activation (44).

LAG-3 is expressed in Treg, activated T cells (both CD4+ and CD8+), and a subset of NKs. This molecule binds to MHC-II due to its similarity with CD4 co-receptor among other receptors in CD8 T cells and NKs (43). Regarding Treg, LAG-3 is essential for immune homeostasis control by Treg, and is necessary for Treg cell-to-cell mediated suppressive mechanism, together with PD-1 and CTLA-4 (41). Similarly to these molecules, LAG-3 supresses cell proliferation, cytokine secretion, and immune function, playing a role in immune homeostasis (42). Expression of LAG-3 confers CD4+ T cell suppressive activity, whilst its blockade on Treg prevents their suppressive functions (43). LAG-3 constitutive expression is associated with CD4+ and CD8+ T cell exhaustion (42). It is also worth noting that LAG-3 is also expressed on Tr1 (type 1 regulatory T cells) although it is not clear whether it is necessary for their suppressive function (43).

**Figure 2:**
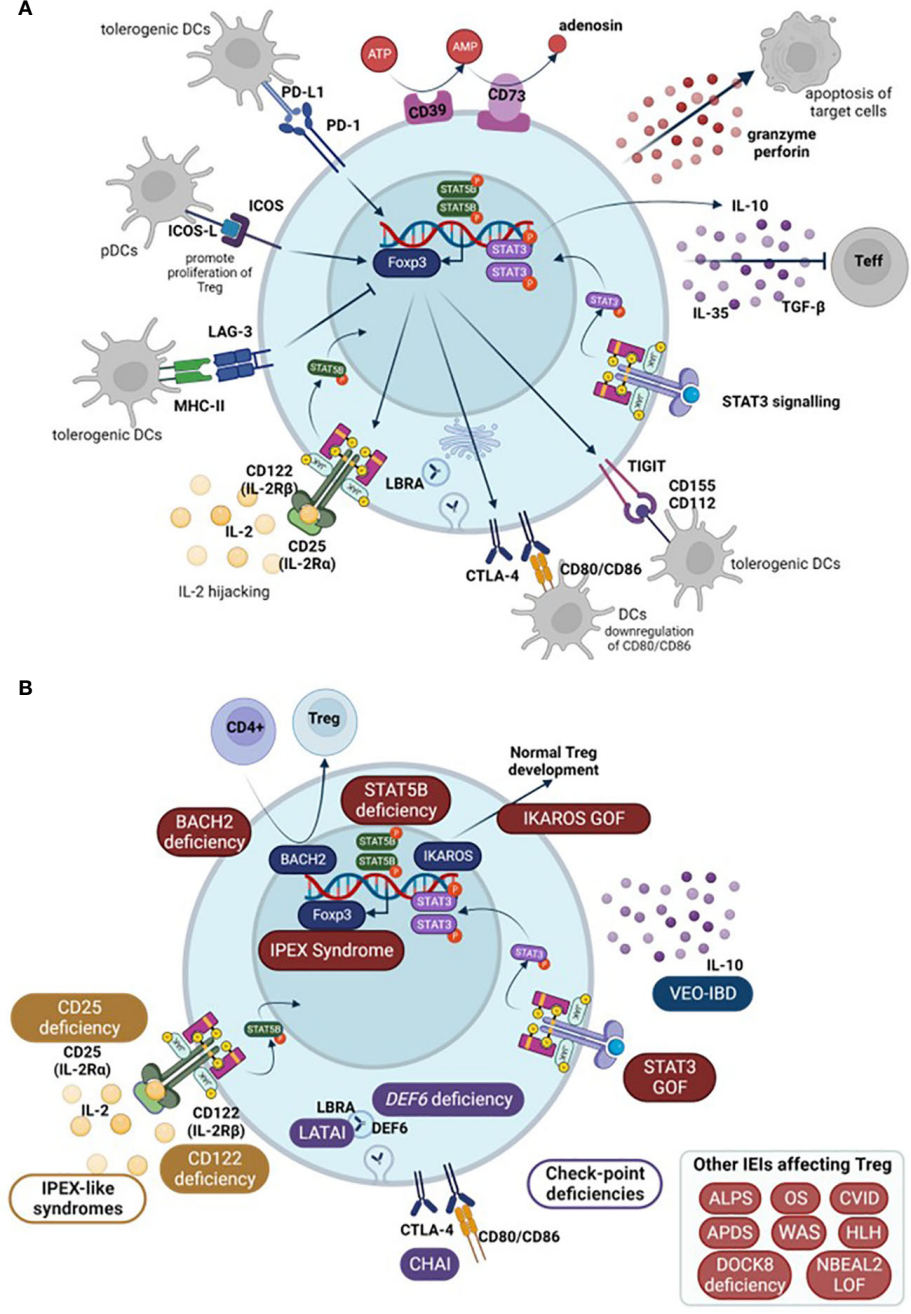
**(A)** Schematic representation of Treg mechanisms of action. **(B)** Main Tregopathies and the gene mutation responsible for the disruption of Treg function among other IEIs that affect Treg (in box, down-right). Arrows show normal physiological processes. Teff, effector T cells; DCs, dendritic cells; pDCs, plasmacytoid DCs; IEI, inborn errors of immunity; IPEX, immune dysregulation polyendocrinopathy enteropathy X-linked syndrome; LATAI, LRBA deficiency with autoantibodies, regulatory T (Treg) cell defects, autoimmune infiltration; CHAI, CTLA-4 haploinsufficiency with autoimmune infiltration; ALPS, Autoimmune lymphoproliferative syndrome; OS, Omenn Syndrome; HLH, hemophagocytic lymphohistiocytosis; CVID, Common variable immunodeficiency; VEO-IBD, very early onset inflammatory bowel disease; APDS, Activated PI3K Delta Syndrome; WAS, Wiskott-Aldrich Syndrome; DOCK8, Dedicator of cytokinesis 8 deficiency; NBEAL2, Neurobeachin-like 2.

Helios is a member of the *IKAROS* transcriptional factor family gene, which has been shown to play an important role in Treg function. It is implicated in Treg maintenance as they ensure FoxP3 stable expression under inflammatory conditions, preventing Treg from becoming effector cells (45). Moreover, in Helios-deficient mice, Tregs fail to produce an appropriate cytokine response and may exhibit the expression of cytokines typically associated with other effector CD4 T cells. Development, function, and immune homeostasis in most CD4+ T cells are not affected. However, in the case of Treg, Helios deficiency, although not affecting development, translates into a late onset autoimmune disease, underlying the importance of this transcription factor in the functionality of Treg (46). The schematic representation of Treg mechanisms of action in humans is summarized in **Figure 2**.

## 2 IEI and Treg: the new kid on the block

### 2.1 From primary immunodeficiencies to inborn errors of immunity to primary immune regulatory disorders

Bruton’s agammaglobulinemia was first discovered seven decades ago. Since then, and thanks to technological advances in recent years such as next-generation sequencing (NGS), whose massive sequencing technology of the human exome and genome has been made affordable worldwide, we have seen a rapid increase in the rate of discovery of new genetic defects in primary immunodeficiency disorders (PIDs). This has allowed experts from the WHO’s International Union of Immunological Societies (IUIS) to collect more than 485 genes causing PIDs by 2022 (47).

PIDs are a group of disorders that affect the proper functioning of the immune system, most of them of genetic origin. Since the immune system protects us against various pathogens, patients with PIDs suffer from a wide range of severe and/or recurrent infections. Although one of the mainstays of immunodeficiency is immunocompromise, which is characteristic of most patients with PIDs, their symptomatology is, nevertheless, increasingly diverse, ranging from autoimmunity, autoinflammation, and severe allergies to neoplastic syndromes (48). The presence of autoimmunity in the context of immunodeficiency suggests a paradox, with two opposite pathologies, where one offers an excess and the other a defect of response, respectively, coexisting in the same clinical entity (49). Due to this increasing phenotype and with the increasing number of genetic defects being discovered, some of them causing gain-of-functions and not only loss-of-functions, the IUIS proposed a paradigm shift in 2019. Since then and to this day, PIDs are known as Inborn Errors of Immunity (IEI), and the classification of these diseases is updated every other year (47, 50).

The latest update of the IUIS in 2022 classifies IEIs into the following 10 groups (47, 51): 1. Combined immunodeficiencies. 2. Combined immunodeficiencies with syndromic characteristics. 3. Predominant antibody deficiencies. 4. Diseases of immune dysregulation. 5. Congenital phagocyte defects. 6. Defects in the intrinsic and innate immunity. 7. Autoinflammatory diseases. 8. Complement deficiencies. 9. Bone marrow failure. 10. Phenocopies of inborn errors of immunity.

Inborn errors of immune dysregulation (group 4) are a heterogeneous group of disorders with clinical variability that affect genes involved in the regulation of the immune system. Those presenting with a phenotype caused by loss of tolerance mechanisms leading to autoimmunity, autoinflammation, lymphoproliferation, and/or severe atopy have come to be recognized as having Primary Immune Regulatory Disorders (PIRD) since 2020 (52, 53). PIRD encompass a diverse set of disorders due to failures in different immune regulatory pathways; hence, this enables a subgrouping into different categories.

### 2.2 From primary immune regulatory disorders to tregopathies: central role of Treg in the pathophysiology

In 2018, the term tregopathies (54) was first introduced: it referred to a group of IEI with a clinical phenotype later encompassed within PIRD, in which the affected regulatory target is the Treg cell itself. This group initially included mutations in *FOXP3*, *CD25*, *CTLA-4*, *LRBA*, *BACH2*, *IL10*, and gain of function (GOF) of *STAT3* (47, 55, 56). Since then, the IUIS expert committee (EC) has added new genes to this category, including mutations in *FERMT1*, *CD122*, *DEF6*, and *IKAROS* GOF (47, 51) (**Figure 2**).

Immune dysregulation, polyendocrinopathy, enteropathy, X-linked syndrome (known by the acronym IPEX) was first recognized in 1982 by *Powell* et al. and was the first disease associated with a defect in Treg. It presents with a wide variety of autoimmune manifestations in early life. IPEX is generated as a consequence of mutations in the transcription factor FoxP3, resulting in a quantitative reduction of protein expression or a loss of protein function (57, 58). However, several studies have shown that the *FOXP3* gene is not essential for the establishment of the Treg cell lineage, but rather for its suppressive effector functions, as it controls the expression of different regulatory molecules such as TIGIT, CTLA-4, and the suppression of the IL-2 gene and different effector T-cell cytokines (59–61). Reported mutations causing IPEX are null or nonsense variants and small deletions or insertions in the reading frame (62–64) in the *FOXP3* gene that result in a residual expression of the protein, but lacking function. Therefore, Treg cells from these patients are dysfunctional, being unable to inhibit the proliferation and inflammatory functions of effector T cells (Teff) (62, 65, 66). Of the 70 mutations identified, 40% are located in the DNA-binding region (FKH), 23% in the proline-rich terminal region (PRR), 9% in the leucine helix (LZ), 14% in the LZ-FKH loop, and 6% in the non-coding region. In addition, human *FOXP3* gene has two main isoforms, a full-length isoform (*FOXP3 FL*) and an isoform lacking part of exon 2 (*FOXP3 ΔE2*) (67). A recent study shows that the lack of exon 2 of *FOXP3* in mice and patients with IPEX syndrome leads to severe immune disorders (67). Even though Treg with *FOXP3 ΔE2* can suppress T cells in culture, *in vivo* they lose their stability, suggesting that the presence or absence of exon 2 in *FOXP3* is critical for normal Treg function (67). Although these *FOXP3* mutations are linked to IPEX, there is an increasing number of patients who do not manifest with the classical triad of early-onset intractable diarrhea, type 1 diabetes (T1D), and eczema. Instead, these atypical cases of IPEX manifest with late-onset of symptoms, single-organ involvement, and mild disease phenotypes (68). Thus, a better understanding of the connection between the genotype and phenotype of this disease is needed.

A variety of tests have been developed to assess key molecules, signaling pathways, and cells implicated in IPEX or IPEX-like diseases for diagnostic purposes. Some are clinically available, while others are only available as research tests. On the one hand, Treg levels and FoxP3 expression can be assessed by flow cytometry. For this methodology, it is common to stain cells with CD4/CD25/CD127 to assess Treg cell frequency or numbers (69, 70). In the European Society for Immunodeficiencies (ESID) registry, working definitions for the clinical diagnosis of IEI (71) are used only for patients with no genetic diagnosis. FoxP3 expression by CD4+CD25+ on flow analysis appears as a diagnostic criterion in IPEX and IPEX-like suspected disease. Nevertheless, patients with nonsense mutations have normal FoxP3 frequency but typically decreased median fluorescence intensity (MFI) because of reduced expression levels (55). Thus, the clinical utility of this technique is moderate (55). The suppressive capacity of Treg can also be measured *in vitro* through a T-cell coculture suppression assay, but this requires cell separation with a significant blood volume. In addition, there are no reference values for this assay yet, so the clinical utility is poor. A clinically useful test is the assessment of IL-2 signaling *in vitro* through T-cell proliferation in the presence of mitogens or IL-2. Mutations in either IL-2 receptor chains will lead to decreased proliferation.

It is also considered useful to measure IL-10 signaling through studies of STAT3 phosphorylation following IL-10 stimulation. Mutations in the IL-10 receptor lead to a loss of STAT3 phosphorylation (55). IL-10 enhances iTreg differentiation and function probably through the STAT3 and Foxo1 signaling pathway (72). Inhibition of Foxo1 blocks the ability of IL-10 to enhance iTreg differentiation and function, suggesting that the effect of IL-10 is dependent on Foxo1 activation (72). This finding relates to the well-known role of Foxo1 as a contributing factor to Treg function and development (73, 74). In addition, IL-10-induced inhibition of STAT3 phosphorylation also reduces the ability of IL-10 itself to enhance iTreg differentiation, suggesting that STAT3-defective CD4+ T cells respond poorly to IL-10, contributing to immune dysregulation (72).

CD25 deficiency is an immune dysregulation disorder segregating in autosomal recessive form caused by biallelic variants in the *IL2RG* gene encoding IL-2Rα also known as CD25 protein. CD25 is expressed at high levels by CD4+ CD25+FoxP3+ Treg cells and has a pivotal role in Treg since IL-2 (and CD25) is required for IL-10 production, as mentioned above (75). Patients with CD25 deficiency present with a clinical phenotype overlapping that of IPEX patients, thus being termed as having IPEX-like disease (75). CD25 expression on the T-cell surface is completely abolished in all patients described, enabling diagnosis by immunological phenotyping with flow cytometry. Decreased T-cell proliferative responses to non-specific or antigen-specific stimuli were observed in some patients, although this could be restored with high doses of IL-2 or IL-15 in some of them. In addition, patients tend to have normal or reduced counts of FoxP3+ regulatory T cells, which makes their isolation for functional studies difficult (75). However, recent studies have demonstrated an altered suppressive capacity in CD25-deficient regulatory T cells purified as CD4+TIGIT+CD127- T cells (76).

In humans, CTLA-4 haploinsufficiency causes a dominant immune dysfunction known as CTLA-4 haploinsufficient autoimmune infiltrative disease (CHAI). Patients with CHAI experience autoimmune cytopenia, hypogammaglobulinaemia, infectious and non-infectious lung disease, enteropathy, lymphoproliferation, skin conditions and neurological involvement, and increased infectious susceptibility. In addition, patients with CTLA-4 haploinsufficiency have decreased numbers of circulating B cells and memory B cells, as well as NK and T cells. In some cases, an increase in CD4+ helper T cells is observed. It is important to note that Treg analysis is made challenging by the low CD25 expression observed in many CVID patients (77). Thus, although the expression of Foxp3 and CD25 may be decreased in CTLA-4 and LRBA deficiencies, this may not necessarily indicate a low Treg count (78). CTLA-4 deficient Treg cells also show decreased suppressive capacity, as in patients with IPEX syndrome (79–81).

LRBA deficiency, either homozygous or compound heterozygous, causes CTLA-4 deficient expression on cell surface. It is also known as LRBA disease with autoantibodies, Treg cell defects, autoimmune infiltration, and enteropathy (LATAI disease), and it shares phenotypic and clinical features with CTLA-4 haploinsufficiency. The CTLA-4 protein is constantly recycled between the cell surface and the cell interior. LRBA facilitates the recycling of CTLA-4 from endosomes to the surface of T cells to prevent its degradation in lysosomes. As a result, LRBA-deficient Treg cells show reduced levels of CTLA-4 expression. Similar to *CTLA-4* mutated patients, LRBA-deficient T cells are hyperproliferative upon *in vitro* activation and both Treg cell-mediated suppressor capacity and CD80 transendocytosis are impaired. Also, Treg cell counts are reduced (82, 83).

A very recent study has revealed the interaction of the Neurobeachin-like 2 (NBEAL2) protein with immune cells (84). Loss of function of NBEAL2 leads to grey platelet syndrome (GPS) and some patients develop autoimmune disorders (84). By mass spectrometry it was shown that one of the proteins associated with NBEAL2 is LRBA. Immunoprecipitation further confirmed that NBEAL2 and CTLA-4 interact with each other (84). Interestingly, NBEAL2 deficiency leads to low CTLA-4 expression in Tconv cells, while Treg cells appear to be unaffected (84). Therefore, further studies of this deficiency are needed to elucidate both phenotypic and functional involvement of Treg cells.

BACH2 is a transcription factor that plays a crucial role in B-cell class recombination, somatic hypermutation, T-cell differentiation and function, and alveolar macrophage function. Genetic variations at the *BACH2* locus are commonly associated with an increased risk of various autoimmune and inflammatory diseases, such as rheumatoid arthritis, T1D, asthma, multiple sclerosis, vitiligo, Graves’ disease, Crohn’s disease, and celiac disease. In 2017, BACH2 haploinsufficiency was initially described in a patient with lymphocytic colitis, splenomegaly, and progressive humoral deficiency with sinopulmonary infections (85). With further cases reported, the disease is now named BACH2-related immunodeficiency and autoimmunity (BRIDA). Patients have been shown to exhibit decreased levels of Treg in the blood and colon (85), where FoxP3 expression levels decrease, compromising Treg (85). Further functional studies are needed to elucidate the role of Treg in this disease.

The STAT3 signaling cascade plays an important role in the regulation of Treg and Th17 cells. STAT3 gain-of-function (GOF) syndrome is a multi-organ primary immune regulatory disorder characterized by early onset autoimmunity. Early in life, patients present most commonly with lymphoproliferation, autoimmune cytopenias, and growth delay (86). STAT3 induction by IL-6, together with TGF-β, is essential for Th17 cell differentiation. However, IL-6 also inhibits TGF-β-induced upregulation of FoxP3 in naïve CD4+ T cells, favoring polarization towards a potentially pathogenic Th17 phenotype (87). In addition, STAT3 activation increases the expression and secretion of SOCS3 (STAT5 inhibitory protein), a positive regulator of FoxP3 and CD25. STAT3 GOF disease may result from altered upregulation of both FoxP3 and imbalance in Treg/Th17 cell polarization, although it is unclear which factor has a dominant effect. Thus, Treg cell counts are usually reduced in STAT3 GOF (88). Because of the broad role of STAT3 in regulating various signaling, differentiation, growth, and regeneration cascades in different tissues and cell types, it is possible that other cell subsets and pathways also contribute to immune dysfunction in these patients (88).

The disturbances in Treg in Tregopathies are summarized in **Table 2**. Currently there are overlapping terms to refer to some PIRD disorders: some are based on physiopathology such as Tregopathies (53) or check-point deficiencies (81, 97), while others refer to the clinical phenotype of affected patients: autoimmune lymphoproliferative syndrome (ALPS)-like (92) and IPEX-like (66).

**Table 2:** Summary of published information on Treg numbers and function in IEI.

Treg numbers and function in IEI				
Tregopathies				
Disease	Gene	Treg phenotype	*In vitro* Treg function	Reference
IPEX syndrome	FOXP3	Normal or low circulating FoxP3+ Treg and FoxP3 expression level; high TSDR demethylation	Decreased suppressive capacity	(55)
CD25 deficiency	IL2Rα	Normal or low circulating FoxP3+ Treg	Altered suppressive capacity	(75)
CTLA-4 deficiency	CTLA-4	Normal or high circulating FoxP3+ Treg and low in FoxP3 expression level	Decreased suppressive capacity	(80)
LRBA deficiency	LRBA	Normal or low circulating FoxP3+ Treg and FoxP3 expression level	Altered suppressive capacity	(83)
NBEAL2 deficiency	NBEAL2	NFS	NFS	(84)
BACH-2 deficiency	BACH-2	Low FoxP3 expression level on Treg	NA	(85)
IL-10/IL-10R deficiency	IL10 IL10Rα IL10Rβ	NFS	NFS	NFS
STAT3 GOF	STAT3	Normal or low circulating FoxP3+ Treg	Altered suppressive capacity	(86)
FERMT1 deficiency	FERMT1	NA	NA	NFS
CD122 deficiency	IL2Rβ	NA	NA	NFS
DEF6 deficiency	DEF6	NA	NA	NFS
IKAROS GOF	IKZF1	NA	NFS	NFS
Other Inborn Errors of Immunity				
HLH	PRF1 UNC13D STX11 STXBP2 SLC7A7 CDC42 FAAP24 RHOG LYST RAB27A AP3B1 AP3D1 SAP XIAP CD27 CD70	Low circulating FoxP3+ Treg	NFS	(89)
ALPS	TNFRSF6 TNFSF6 CASP10	Low circulating FoxP3+ Treg; high proportion of naïve Treg (FoxP3lowCD45RA+)	Altered suppressive capacity	(90)
VEO-IBD	XIAP	Normal or low circulating FoxP3+ Treg and FoxP3 expression level	Altered suppressive capacity	(91)
APDS	PIK3Cδ PIK3R1	Low circulating FoxP3+ Treg	Altered suppressive capacity	(92)
CVID	Unknown	NFS	NFS	NFS
WAS	WASp	Low circulating FoxP3+ Treg	Normal or slightly lower suppressive capacity	(93)
DOCK8	DOCK8	Low circulating FoxP3+ Treg	Altered suppressive capacity	(94)
OS	RAG1 RAG2 DCLRE1CIL7R RMRP IL2-Rγ ZAP70 LIG4 ADA	Low circulating FoxP3+ Treg and FoxP3 expression level. Severe reduced FoxP3+ Treg in thymus	Reduced suppressive capacity	(95)
STAT5b	STAT5b	Low circulating FoxP3+ Treg and FoxP3 expression level	Altered suppressive capacity	(96)

NFS, need further studies; NA, not available.

### 2.3 Treg dysfunction in other IEI

The remaining categories of PIRD which are not recognized as Tregopathies include hyperinflammatory disorders, such as hemophagocytic lymphohistocytosis (HLH) with or without intrinsic susceptibility to Epstein-Barr virus, non-malignant lymphoproliferation with autoimmunity (ALPS), inflammatory bowel disease linked to IL-10 signaling (very early onset inflammatory bowel disease, VEO-IBD), and other disorders linked to monogenic autoimmunity but lacking a central role in Treg (51, 53). Although the genetics of these other PIRD do not impact directly on the biology and function of Treg, these cells can be markedly altered in a direct or indirect manner.

HLH is a hyperinflammatory disorder with excessive immune activation resulting from different genetic defects in *PRF1, UNC13D, STX11, STXBP2, SLC7A7, CDC42, FAAP24, RHOG, LYST, RAB27A, AP3B1, AP3D1, SAP, XIAP, CD27, or CD70* (51, 98) that impair granule-mediated cytotoxicity. This impedes the cytotoxic effector cells from achieving their goal: to kill infected and transformed cells, with the consequent release of cytokines that promote a state of hyperactivation of the immune response and hyperinflammation. It has been shown that in acute HLH CD8+ T lymphocytes overexpresses CD25, so that by consuming IL-2 from the medium they compete against Treg for this very resource. This results in an indirect Treg cell malfunction and a collapse in Treg cell numbers (89). In the specific case of X-linked inhibitor of apoptosis protein (XIAP), which causes X-linked Lymphoproliferative Disorder (XLP-2) and VEO-IBD, Treg cells are also affected. Suppressor of cytokine signaling 1 (SOCS1) is one of the transcription factors involved in the maintenance of Treg inhibitory function. XIAP binds to SOCS1 promoting its stabilization (99). Mutations in XIAP lead to SOCS1 deficiency and trigger an overactivation of the transcription factors STAT1 and STAT3 with a consequent reduction of the suppressive capacity of Treg, reducing their cell numbers, reducing FoxP3 stability, and even predisposing to IFNγ secretion, which diverts the response from a regulatory function to an inflammatory one (91).

In the case of ALPS, the underlying genetic defect involves the components of the apoptotic Fas-FasL pathway. There are a number of biomarkers linked to ALPS such as elevated levels of CD3+TCRαβ+CD4-CD8- double negative cells, as well as elevated plasma levels of IL-10, soluble FasL (sFasL), and vitamin B12 (92, 100). It has been shown that in ALPS patients the proportion of Treg defined as both CD25^hi^CD127^low^ and CD25^hi^FoxP3+Helios+ was lower compared to healthy controls (90). In addition, patients also had a high proportion of naïve Treg (FoxP3^low^CD45RA+) and a rare population defined as CD4+CD127^low^CD15s+CD45RA+. However, although the proportions of Treg were low, the suppressive capacity on T-cell proliferation was not affected. This study suggests that excessive T cell proliferation in ALPS may not be ascribed to a Treg defect or T cell sensitivity to suppression (90). However, given the paucity of information on Treg in ALPS, further studies are needed.

Furthermore, in recent years and thanks to advances in NGS, novel gene defects outside the Fas-FasL apoptosis pathway with a phenotype and clinical mimicry to ALPS have been identified (ALPS-like). These include the above-mentioned check-point deficiencies (CTLA-4, LRBA and DEF6 deficiencies) but also the Activated PI3K Delta Syndrome (APDS). APDS results from the increased activity of the phosphoinositide-3-kinase δ (PI3Kδ) pathway (101). This enzyme is predominantly expressed by leukocytes and plays a very important role in immune cell function. Animal models indicate that impaired PI3Kδ in mice leads to colitis mostly due to compromised Treg function, suggesting PI3Kδ plays a role in Treg function (102). However, it should be pointed out that the mouse model of PI3Kδ does not recapitulate ALPS in humans. In most of these ALPS-like disorders, total Treg CD4+CD25+FoxP3+ levels are lower compared to healthy controls, as well as lower CTLA-4 expression levels and an expansion of circulating follicular CD4+CXCR5+CD45RA- (cTFH) T cells and polarization towards a Th1 phenotype (CCR6-CXCR3+) (92). Also, in most ALPS-like phenotypes there is a reduction in the T cell compartment, while senescent CD4+ T cells are increased.

Clinical phenotypes of lymphoproliferation and autoimmunity are found in other entities such as common variable immunodeficiency (CVID). In CVID, the involvement of Treg cells is still under study; recent studies have reported decreases in CD4 Treg in patients with CVID (103), while others have found conflicting results (104). For example, some researchers have observed that CD4 Treg (identified by markers such as CD25^hi^CD127^low^FoxP3+) are decreased in CVID patients, especially those with associated autoimmune diseases (105). However, other studies have found no significant changes in CD4 Treg in relation to disease severity or the presence of autoimmunity (104). Some researchers have also explored the function of these cells and found that, despite differences in levels, the suppressive function of CD4 Treg appears to be maintained overall (106). However, in general, there is still a lack of consensus on how CD4 Treg relate to the pathophysiology of CVID and its association with autoimmunity (103, 107, 108). In addition, a recent study analyzed CD8 Treg (CD8+CD25^hi^CD183+Foxp3+) in patients with CVID, finding significant reductions in CD8 Treg ratios in these patients (109). However, no significant differences in CD8 Treg ratios were observed between patients with CVID with or without autoimmunity (109). Because CVID is an umbrella term for a probable heterogeneous group of diseases with different pathophysiological mechanisms (110), the study of Treg in this disease is challenging. Further research with larger series and more specific approaches are needed to fully understand the role of CD4 and CD8 Tregs in the pathogenesis and clinical manifestations of CVID and its relationship with autoimmunity. The disturbances in Treg in IEI other than Tregopathies are summarized in **Table 2**.

Wiskott-Aldrich syndrome (WAS) is an X-linked IEI characterized by lymphocyte dysfunction leading to opportunistic viral and bacterial infections, thrombocytopenia, eczema, autoimmune disorders, and cancer (93, 111, 112). More than 200 mutations have been described in the gene encoding the WAS protein (WASp) (93, 111). Mutations resulting in loss of WASp expression correlate with a more severe disease phenotype (93). Its deficiency is mainly associated with defects in T-lymphocytes. In particular, lymphocytes are unable to reorganize the actin cytoskeleton in response to T-cell receptor (TCR) engagement, leading to incomplete cell activation, and decreased cell proliferation and survival. Although WASp has not been shown to play a crucial role in the production of Tregs in the thymus, it is required for Treg expansion and survival in the periphery (93, 111). WASp deficiency leads to a decrease in the percentage of peripheral Treg (although FoxP3 expression is maintained), with a strong impact on activated Treg as evidenced by a decrease in activation markers and migration receptors (93, 112). Despite this, WASp-deficient Treg show normal or slightly lower suppressor capacity against T cells, suggesting that the impact of WASp mainly affects the survival and expansion of peripheral Treg, but not their suppressor function (93). Notably, in a study of WASp-deficient mice, Tregs were defective in their suppressor function *in vitro*, but in WAS patients this defect in function was less clear (113). In terms of clinical implications, although alterations in Treg may correlate with the high frequency of autoimmunity in WAS patients, more research is needed to improve understanding of this correlation.

Dedicator of cytokinesis 8 (DOCK8) deficiency is a rare IEI characterized by a constellation of symptoms including severe immunodeficiency, elevated IgE levels, allergies, and autoimmune disorders (94, 114). While the precise pathophysiology of immune dysregulation remains only partially understood, it is suggested that DOCK8 regulates the suppressive function of Tregs by promoting STAT5 phosphorylation in response to IL-2 signaling, which is essential for Treg maintenance (94). Furthermore, DOCK8 is involved in CD25 recycling (94). Patients with DOCK8 deficiency have been shown to exhibit reduced levels of Treg with impaired suppressive function (94). Remarkably, in a study using contact hypersensitivity (CHS) models, Treg lacking DOCK8 acquired a pathogenic phenotype expressing both FoxP3 and T-bet, along with IFNγ, suggesting that Tregs polarized towards a potentially inflammatory phenotype rather than maintaining their suppressive function (94, 114).

Omenn syndrome (OS) is an severe recessive autosomal combined immunodeficiency, ascribed to hypomorphic mutations in the activating recombination genes (*RAG1* and *RAG2*), but also in other genes (*DCLRE1C, IL7R, RMRP, IL2-Rγ, ZAP70, LIG4, ADA*) (115), and impairing, but not abolishing, the recombination process of the *V(D)J* genes. OS manifests with generalized erythroderma, alopecia, lymphadenopathy, hepatomegaly, and diarrhea, and the immunological phenotype is characterized by a reduced number of circulating B lymphocytes (95, 115). In one study, patients with OS were shown to have a variable number of circulating Treg with low FoxP3 expression and reduced or no T-cell suppressive capacity *in vitro*, together with a severe reduction of Treg cells in the thymus (95). These results provided the first evidence of a defect in Treg development and function in OS, implying that both central and peripheral tolerance are compromised in this disease.

STAT5b deficiency is a rare autosomal recessive disease involving both marked growth failure and severe immunodeficiency (116). Normal transcription of *IL-2Rα, FOXP3, Bcl-2* and growth hormone (GH) genes are controlled by STAT5b signaling (96, 116). Immunophenotypically, STAT5b-deficient patients have decreased Treg numbers, low expression of FoxP3 and CD25, and an impaired ability to suppress T-cell proliferation (96, 116). Patients present with severe eczema, arthritis, autoimmune thyroiditis, and thrombocytopenic purpura, which are thought to be associated with Treg dysfunction (96, 116), demonstrating that STAT5b plays a critical role in Treg maintenance and function. Interestingly, an overshadowed cytokine, IL-9, activates the transcription factors STAT3 and STAT5 whose cellular targets include Treg (117). In an IL-9Rα-deficient mouse model of autoimmune encephalomyelitis, Tregs have a reduced ability to suppress T-cell proliferation (117). In addition, *in vivo* and *in vitro* assays have shown that IL-9 promotes Treg suppressive function and survival through STAT3 and STAT5 signaling (117). This suggests that IL-9 has an important role in Treg function and maintenance. Therefore, it could be of interest to include the study of IL-9Rα or IL-9 function in Treg in patients with immune dysregulation immunodeficiencies.

## 3 Treg cell-based therapies

The diagnosis and management of IEIs pose challenges due to the confluence of overlapping and multisystemic manifestations and their variable underlying genetic etiologies (54). Also, there is a variety of treatment options for the management of immune dysregulation in IEIs depending on the specific type, severity, and pathway involved in the disorder. These include systemic immunosuppressants, enzyme replacement therapy, small molecules, biological therapies (such as monoclonal antibodies), and adoptive cell therapies (ACT) such as hematopoietic stem cell transplantation (HSCT), gene therapy (54, 118, 119) and, in the near future, cell-based therapies. The aim of this review is to ascertain available Treg cell-based therapies more comprehensively and, as such, the other therapies will be briefly summarized.

### 3.1 Current therapeutic approaches for immune dysregulation in IEI

Systemic immunosuppressants, such as glucocorticoids and calcineurin inhibitors, exhibit non-specific effects and are typically employed in cases where specific diagnosis is still lacking, but there is a requirement to manage inflammation (54, 118). As for targeted therapies, there is a range of pharmaceutical agents with the ability to selectively counteract the aberrant pathways and/or molecules involved in specific diseases, thereby tailoring drug-based therapies to the precise needs of each individual patient. Within these possibilities, monoclonal antibodies are used as a more personalized approach to treat some IEIs. This is the case of alemtuzumab (anti-CD52) (56), emapalumab (anti-IFNγ) (120, 121) and anakinra (IL-1R agonist) (122) used to treat HLH. Other drugs include rituximab (anti-CD20) used for autoimmune and lymphoproliferative disorders (121) and tocilizumab (anti-IL6R) for GOF STAT3 (123, 124). Moreover, a recent clinical trial explored the efficacy and safety of tadekinig alpha (an IL-18 binding protein) in autoinflammatory conditions driven by IL-18 (Arnold) such as *NLRC4* GOF and XIAP deficiency (125). Regarding IL-10 related VEO-IBD, IL-1 inhibitors have been suggested based on the assumption that IL-10 LOF mutations can interact with activation through the inflammasome which would lead to increased IL-1 levels (126). Abatacept (CTLA-4 fusion protein) is used in CTLA-4 and LRBA deficiencies (127). Additionally, due to its mechanism of action inducing T cell anergy it has been tested in a mouse model for IPEX with very promising results and superior efficacy to sirolimus and anti-CD4 treatments (128). Teplizumab, a CD3-directed monoclonal antibody, has been approved to treat T1D and is being tested in pediatric patients with stage 2 of this disease (129). Considering the occurrence of T1D in IPEX patients, this therapeutic approach could be highly beneficial in effectively managing these patients (63). Besides monoclonals, other drugs targeting specific affected pathways are being used. For example, sirolimus (an mTor pathway inhibitor) has been used in LRBA biallelic mutations and CTLA-4 haploinsufficiency (56), as well as ALPS and APDS (120) and CVID (130). This is also the case for Jakinibs (direct JAK/STAT inhibitors) like ruxolitinib (JAK1 and JAK2 inhibitor) and tofacitinib (JAK1 and JAK3 inhibitor) that have been used in *STAT3* GOF mutations (56, 120) and HLH (131).

HSCT is a curative option for IEIs. Within the context of IEIs, the initial application of HSCT was documented in SCID cases as early as 1968 (120, 132). This procedure carries substantial risks, including graft-versus-host disease (GVHD) and the administration of conditioning agents that result in grave immunosuppression, thereby elevating the risk of severe infections (54). Furthermore, limitations encompass the availability of compatible donors, the age of patients, and the severity of the disease and/or end-organ damage (118). Due to the importance of conditioning regimes for survival, HSCT recommendation guidelines are established by the EBMT/ESID inborn errors working party guidelines (IEWP) (118, 126, 133). PIRD has particular challenges for HSCT. This approach becomes essential in patients that cannot be managed by immunosuppressive treatments (125). In this context, patients show an increased risk of alloreactivity to HSCT indicating a need for further research into the use of targeted treatment as a bridge for HSCT (134). Moreover, while this approach has demonstrated efficacy in resolving clinical symptoms for a subset of patients, the long-term survival rates remain relatively low compared to conventional IEIs. For PIRD, the survival rate at the 5-year mark is approximately 67% (135) whilst conventional IEIs exhibit a 90% (134). Regarding HSCT in PIRD, even when the donor is a close relative, genetic screening becomes necessary due to the disease’s delayed and variable clinical onset or incomplete penetrance, which could also potentially impact other family members (134). Therefore, comprehensive research is imperative to render HSCT in PIRD a safer, more efficient and viable therapeutic approach.

Gene therapy was first used in SCID-ADA as early as 1993, followed by SCID-X1 (132). This strategy entails the modification of HSC and other immune cells involved in the disease process. Subsequent to its inception, significant advances have been made such as using self-inactivating lentiviruses, rendering this approach a promising and safer alternative compared to its earlier iterations (119, 120). Indeed, in 2016, Strimvelis™ was approved in Europe for the treatment for ADA-SCID. It consists of autologous CD34+ cells (HSC) transduced with a gamma-retrovirus that encodes for the human ADA cDNA sequence. There are, however, important limitations for this gene-adding approach. Transduction can limit the success and number of cells available for treatment, and gene manipulation can generate insertional mutagenesis that can turn on oncogenes and lead to clonal expansion or leukemic proliferation. Moreover, preexisting mutations or availability of autologous cells for gene modification could rule out the patient for the procedure (136). A more pinpointed approach is gene editing where stable genetic modification is introduced at the specific targeted site that is mutated and responsible for the IEIs. This process employs engineered nucleases, with three primary types being utilized: Zinc-Finger Nucleases (ZFNs), Transcription activator-like effector nucleases (TALENs), and CRISPR/Cas. These molecular tools enable researchers to precisely edit the genetic material, offering a more targeted and efficient approach to treating genetic-based immune disorders (119). This technique is still in preclinical phases but demonstrates remarkable promise. Regarding gene therapy in PIRD, is has been evaluated in IPEX syndrome and HLH. In IPEX, two primary gene therapy strategies are currently under development. The first consists in forcing ectopic and stable FoxP3 expression on CD4+ T cells, thereby transforming them into FoxP3 engineered Treg-like cells. This approach is already being tested in IPEX patients where a clinical trial (NCT05241444) is evaluating the feasibility and safety profile of these Treg-like cells, called CD4^LVFoxP3^. The second approach, which is still in preclinical studies, uses CRISPR-Cas to modify hematopoietic stem and progenitor cells (HSPCs) for the development of long-lasting and functional Treg and Teff cells that can normally express FoxP3. This latter approach is capable of restoring FoxP3 levels independent of the specific mutation of the patient and it also enables cell marking for monitoring and purification purposes (137). In HLH, there is also a gene therapy approach under development for perforin deficiency (138). It consists in transducing autologous murine CD8+ T cells with a gammaretroviral vector with corrected perforin gene to generate fully functional CD8+ T cells, which are subsequently transferred back to the mouse. Indeed, this strategy has demonstrated success *in vivo* in an HLH murine model (139). These promising findings offer hope for the potential application of gene therapy in the treatment of IPEX and HLH in the future.

### 3.2 Adoptive cell therapy with Treg

With the first human treated with Treg in 2009, ACT using Treg is a rapidly expanding field that offers substantial advantages and holds great promise for medical applications (140). Numerous clinical trials are currently being carried out with Treg cell-based therapies to manage GVHD (39), T1D (141) and transplant rejection (142). As highlighted throughout this review, Tregs exhibit suppressive and immunoregulatory functions that render them ideal for addressing diseases characterized by immune activation. Nevertheless, care must be taken when employing Treg cell-based therapeutic approaches, which are summarized in **Table 1**. Firstly, Treg isolated from blood is a mix of pTreg and tTreg, and tTregs have been shown to be more stable under lymphopenia conditions in mouse models. Secondly, Tregs possess the ability to modulate their local microenvironments, enabling them to convert their surroundings into a suppressive milieu regardless of their own persistence. This phenomenon is referred to as infectious tolerance. Consequently, infused Treg may not necessarily need to endure indefinitely, but rather, they can persist as long as it takes for the microenvironment to undergo transformation and establish a suppressive environment. Furthermore, it is important to consider that when Tregs for therapy are derived from Teff, they may exhibit different mechanisms of action compared to their natural counterpart (37). Numerous studies have demonstrated that antigen-specific Tregs are more efficient and safer than polyclonal Tregs as they migrate to the specific site where the antigen itself is expressed, reducing the risk of general immunosuppression. However, antigen-specific Tregs are present in very low numbers in blood, and the expansion protocol needed for their use as ACT is very challenging (38). Therefore, polyclonal Treg could be a more feasible approach although it could generate more side effects due to bystander suppression (143).

Taking all these aspects into account, various mechanisms are being explored to generate antigen-specific Treg. One method involves the use of an engineered T cell receptor (TCR), where an antigen-specific TCR is obtained from a Teff and introduced into Treg. These transduced cells gather in target tissues expressing the specific antigen, exerting their function directly or by bystander suppression. Importantly, they are MHC restricted so matching MHC between donor and recipient is needed (143). This technique has demonstrated remarkable potential in mouse models of multiple sclerosis and hemophilia, exhibiting promising results (37). Another approach involves chimeric antigen receptor (CAR) Treg cells, which are designed to recognize specific proteins. This strategy proves particularly useful for conditions involving tissue destruction. Compared to engineered TCR Treg, CAR Tregs require high antigen expression to activate but are MHC independent which makes them a more versatile option for widespread application in patients. In fact, engineered CAR Treg against HLA-A2, commonly mismatched in transplantation, have been proven to protect from GVHD in different mouse models (143). A third approach involved overexpressing FoxP3 in antigen-specific Teff. By transducing FoxP3 into CD4+ Teff, stable and heightened expression of this transcription factor is achieved, effectively converting effector T cells into functional Treg cells (37, 144). Similarly, the most frequently used approach in Treg based therapies comprises the obtaining of Treg cells from peripheral blood and stimulating them with anti CD3/CD28 beads along with high IL-2 concentration to achieve a significant expansion of polyclonal Treg. Although this expansion yields a large number of cells, the expanded Treg cells are less specific and potent than antigen-specific Treg (37). An important limitation in approaches that use peripheral blood as the Treg source is the low number of Treg cells that can be obtained, making it challenging to achieve sufficient cell numbers for therapy. Additionally, the expansion process itself can lead to an exhausted phenotype of the Treg, potentially impacting functionality, stability, and efficacy (39).

An exceptionally innovative recent approach consists in generating effective Treg from pediatric thymus, which is routinely discarded in pediatric cardiac surgeries. Referred to as thyTreg, these cells are derived from fresh thymocytes and are induced to differentiate and expanded under controlled laboratory conditions. Notably, thyTreg show highly advantageous properties as they are immature with an undifferentiated phenotype, have greater suppressor capacity, and display increased stability of FoxP3 (39). Given the promising characteristics, thyTreg are currently being evaluated in a clinical trial to prevent rejection in heart transplant patients (NCT04924491). If this therapy succeeds, thyTreg could be further developed for allogenic use as a potentially promising therapy for some PIRD.

There are different methods for obtaining Treg for ACT, generally involving isolation of specific cells from elected source, expansion, characterization of the resulting cell product, reinfusion, and monitoring of the infused cells (38). Throughout this section, different sources for Treg obtention have been mentioned. These sources are continuously explored and allow the isolation of other immune cell types in addition to Treg. These include peripheral blood and umbilical cord blood, and, more recently, Treg cells directly derived from the thymus (where T cells are developed). Each source has its own advantages and challenges, and ongoing research aims to optimize the selection and utilization of these sources to enhance the efficacy and safety of Treg cell-based therapies (39).

### 3.3 Adoptive cell therapy with Treg in PIRD

IPEX syndrome, the benchmark for studying Treg function, highlights the potential benefits adoptive Treg cell therapy could have in IEIs. Indeed, the treatment of the equivalent phenotype in mice, known as scurfy, has shown promising results, leading to an increased lifespan of diseased mice (144). CD4+ T cells were isolated from scurfy mice and transduced with a lentiviral vector containing the whole *FOXP3* transcript, as well as the transcript encoding a truncated surface-expressed protein (CD271), facilitating their selection and further detection *in vivo* (145). Resulting CD4^LVFoxP3^ cells were expanded *in vitro*, resulting in a pool of CD4+ T cells with FoxP3 expression that showed both suppressive function and sustained stability of FoxP3 (137). Initially achieved in scurfy mice, this accomplishment was subsequently extended to encompass CD4+ T cells sourced from IPEX patients (63). This approach demonstrated the feasibility of appropriately transducing previously affected T cells from IPEX patients, characterized by a FoxP3 mutation, resulting in the conversion into CD4+ FoxP3+ T cells. Moreover, these CD4^LVFoxP3^ exhibited the same functional traits as naturally occurring Treg, including suppressive functions and expression of CD25^hi^ CD127^lo^ CTLA-4+ ICOS+ Helios+ (137). Indeed, upon infusion of CD4^LVFoxP3^ cells into scurfy mice, notable immunomodulatory effects were observed, leading to symptom improvement (146).

These findings offer valuable insights into the potential therapeutic applications of Treg cell-based interventions for immune dysregulation disorders such as IPEX syndrome (144). To our knowledge, IPEX syndrome remains the only IEI subject with comprehensive investigation aimed at the development of a Treg cell-based therapeutic approach aimed at curing the disorder.

## 4 Discussion

### 4.1 Changes and controversies in the phenotypical definition of Treg

Treg cells have a unique phenotypic molecular signature characterized by high expression of CD25 and FoxP3, both in tTreg or pTreg (14). Although in mice the expression of Helios and low expression of Neuropilin 1 (Nrp-1) are good molecular discriminants between tTreg and pTreg cells (30, 147), in humans this discrimination is problematic and somewhat unclear. In human tTreg, Helios expression is not universal and, similar to FoxP3, its expression has been reported in activated T cells (148, 149). FoxP3 and Helios provide information about the stability and suppressive power of the cell (150) with Helios+ Treg having been reported to be more stable and with a greater suppressive capacity (151). In mice, Nrp-1 expression indicates greater Treg stability; however, in humans, this protein is virtually undetectable in peripheral Treg (152). Because of this, the use of Helios as a differentiating marker between tTreg and pTreg is a matter of ongoing debate. Nevertheless, because of the information it provides, measuring Helios along with FoxP3 should be included as a routine marker in laboratories whenever possible (153).

Characterization of Treg by flow cytometry may not be so simple. Firstly, and as mentioned earlier, there is no specific unique marker for Treg. Secondly, the relatively low expression of FoxP3 requires the use of fluorochrome-labelled monoclonal antibodies of sufficient intensity to detect it. Within Treg, the degree of FoxP3 expression shows a dynamic range from naïve or resting Treg cells, with low expression, to activated Treg with higher expression (154, 155). Therefore, separate identification among Tconv, resting Treg (rTreg), naïve Treg, and activated Treg would be achieved by the relatively high expression of FoxP3 and by additional markers such as CD45RA, thus defining rTreg as CD45RA+FoxP3^low^ activated Treg cells with suppressive activity (aTreg) as CD45RA-FoxP3^hi^ and T cells without inhibitory capacity as CD45RA-FoxP3^low/-^ (155).

Further complicating the matter, intracellular staining requires a fixation process. Although some molecules, such as formaldehyde, largely preserve the native conformation of proteins, they can also generate cross-linking between proteins, polysaccharides, and lipids that may hide the reactive epitopes for fluorochrome-labelled antibodies (150). Transcription factors are not suitable for the isolation of viable cells, and they require more sample handling, time, and resources, reinforcing the clear need to identify unique surface markers for Treg characterization. One possible solution found through omics analysis is a transmembrane protein known as GPA33 that is expressed by a set of Treg with tTreg-like characteristics (156–158). Additionally, TIGIT appears to inhibit the activation of the mammalian mTORC1 target of rapamycin signaling pathway, maintaining the stability and expression of FoxP3 in Treg (159). Also, elevated TIGIT expression in tTreg (CD4+FoxP3+Helios+) has been reported to be associated with cell lineage stability and suppressive capacity (160). In conclusion, a possible alternative to the use of Helios and FoxP3 as markers of lineage, cell stability, and suppressive force would be the replacement of these markers with GPA33 and TIGIT, respectively. However, further studies are needed to clarify their validity for the identification of stable human Treg.

Given all this information, a crucial question is how human Treg should be defined. Concerning CD4+ cells, there are several ways to define them, either as CD25^hi^CD127^low^, CD25^hi^FoxP3+, FoxP3+, CD25^hi^CD127^low^FoxP3+ or CD25^hi^CD127^low^CCR4+ (69, 70). At present, Treg cells are characterized in the literature as any of the above definitions, therefore emphasizing the need for a consensual and unified characterization to study this cell type. Based on current knowledge, the most rigorous way to define them is as CD25^hi^CD127^low^FoxP3+Helios+, which ensures the selection of tTreg with stable FoxP3 expression. However, as discussed above, the use of FoxP3 and Helios is not possible when working with viable cells, and sometimes all four markers cannot be used together due to instrumentation limitations. If this is the case, at least two of them should be used, either as CD25^hi^CD127^low^ or CD25^hi^FoxP3+ (69, 70). Using each marker separately is not a valid option, as Tconv cells may overexpress CD25, FoxP3, and Helios, and reduce CD127 expression under stimulation conditions (154, 161, 162). CCR4 is currently recognized as a Treg effector cell marker, and therefore can be used to complete the phenotypic definition of Treg when used alongside the markers already mentioned (29).

The complexity of characterizing Treg increases if CD8+ regulatory cells are to be studied, given their recent role in viral infections and tumorigenesis (22). CD8+ Treg can be divided into different groups, including CD8+CD103+, CD8+CD122+, CD8+CD28-, and CD8αα+HLA-E+TCRαβ+ cells (163). CD8+CD103+ Treg have been implicated in studies of kidney transplantation rejection and intestinal graft-versus-host disease (164). TGFβ is directly related to the level of CD103 (αE7β integrin) expression, and it is thought that its suppressive mechanism is through cell-cell contact. However, other studies show that, under inflammatory conditions, CD8 Treg releases TGF-β conferring a protective effect (31, 165, 166). Although these cells are CD28+ and do not express FoxP3, CTLA-4, GITR, LAG-3, or CD25, a stable human CD8+ regulatory type (hCD8+) can be induced by adding TGF-β and Rapamycin (RAPA) together with αCD3/CD28 and IL-2 *in vitro* expressing FoxP3, CD103, and PD-1 (167). Nevertheless, further studies are needed to determine the differences among these cell types.

CD8+CD122+ Treg cells have been shown to exert suppressive function in transplantation and autoimmunity settings (32, 168), especially within the memory compartment (CD44^hi^CD62L^hi^CCR7+CD127-). However, CD8+CD122+ cells do not express FoxP3, suggesting that they are part of a different group from CD8+FoxP3+ (hCD8 Treg) induced CD8+ cells (169–171). A comparative study showed that a subgroup of CD8+CD122+ Treg cells expressing PD1 (CD8+CD122+PD-1+) versus CD4+FoxP3+ Treg cells are more effective in the context of allograft rejection, with this protein being mainly responsible for the inhibitory effects (33, 170). In spite of this, PD-1 is a marker of T-cell exhaustion and functional impairment, so PD-1 expression on CD8+ Treg requires additional research (172).

Other populations have also been recognized as regulatory cells. These include CD8+CD28- cells which have been associated with different clinical conditions such as pregnancy, infectious diseases, cancer, and organ transplantation. Several studies have shown that their suppressive capacity is dependent on IL-10 or TGF-β (34, 173). Although some research groups have demonstrated FoxP3 expression in this cell type (174, 175), other groups have not confirmed this (176, 177). Similarly, loss of FoxP3 and CD28 in this cell type correlates with the expression of CD56, granzyme A, and perforin exhibiting cytotoxic activity (178). In addition, a unique phenotype of CD8αα+TCRαβ+Qa-1 restricted Treg cells (in mice) and HLA-E in humans, expressing surface molecules typical of NK cells, has been demonstrated (35), as well as the recent discovery of a CD8+KIR+ Treg cell subpopulation, which appears to play an important role in the maintenance of peripheral tolerance in the context of antigen-specific viral infections and its relationship to autoimmunity (179). All these findings are of significant importance for understanding the regulatory mechanism of CD8+ cells, but significant further work is needed to clarify precise phenotypic identification, and the possible relationship with CD4+FoxP3+Helios+ Treg.

Currently, the reliable identification of Treg requires a panel with different surface markers, together with function analyses. Taking into account the major problems discussed here, dedicated research is needed to improve our understanding of Treg cells biology and the phenotypic plasticity of the cells in different biological and pathological contexts. These phenotypic controversies are summarized in **Table 1**.

### 4.2 Changes and controversies in the functional studies of Treg

Because phenotypic characterization of human Treg is challenging, measuring the suppressive capacity of Treg in artificial *in vitro* systems can additionally be used for Treg characterization. Nevertheless, this functional assay may or may not accurately represent their functionality *in vivo*, and the assays currently used may be misinterpreted. Despite these limitations, if assays are rigorous and carefully controlled, the data obtained provide a reliable indication of the regulatory potential of Treg.

Traditionally, what has been considered the gold standard to date is the measurement of the suppressive capacity of Treg, particularly in relation to inhibition of proliferation of conventional CD4+ and CD8+ T cells. Previously, cell proliferation measurements had certain limitations given the use of radioactive isotopes (3H-tritylated) and the lack of precision in identifying the proliferating cells. Although it is very sensitive and requires a small number of cells, this technique lacks suppression of proliferation and cannot confirm Treg regulatory function (92, 180–182). Consequently, it is useful to measure inflammatory cytokine production in the supernatant in this type of assay because 1) the analysis of cytokines is independent of Treg activity, and 2) Tregs suppress the action of inflammatory cytokines (183). However, a quicker solution is to replace the suppression assay with the measurement of IL-2 or IFNγ mRNA expression, as well as flow cytometric expression of CD69 and CD154 on Tconv cells, as these alternatives can be performed in 5-7h after co-culture with Treg cells (183, 184). Indeed, commercial companies such as Becton Dickinson (BD) already offer kits to determine Treg suppression based on these two surface markers on Tconv cells after culture at the clinical level (185).

Current flow cytometry-optimized assays using fluorescent reactive amines such as CFSE (Carboxyfluorescein succinimidyl ester) are more efficient and accurate. CFSE labelling of Teff allows independent analysis of Treg from effector cells, as well as proliferation-parallel analysis of surface marker expression and cytokine secretion (186). Although to date this technique is widely used, it should be noted that by analyzing cells that have divided at least once, there will be cells with variable numbers of divisions, so this may underestimate the suppressive capacity of Treg. Therefore, a more accurate approach is the use of cytometry analysis software such as FlowJO (TreeStar) as it provides parameters such as proliferation index (average number of divisions), percentage of divided cells (percentage of cells in the initial population that have divided at least once), and division index (average number of divisions a cell has undergone in the initial population). By basing it on cell divisions rather than the total number of cells, the calculation of the division index allows for a more accurate assessment (36).

Although the *in vitro* suppression assay is a powerful tool, the co-culture system is risky and can lead to misleading data or misinterpretation. Hence, the need for rigorous controls. First, titration of cells in the co-culture is essential. Whilst some studies show that high titers of 1:1 or 1:2 (Treg : Teff) are unlikely to occur under physiological conditions, suppression should be detected at ratios as low as 1 Treg:16 Teff (181, 187). It is essential, however, to have a co-culture control of non-Treg : Teff cells under the same conditions to confirm that the suppression is specifically due to Treg. An additional consideration is that Treg cells have cytotoxic function (Treg CD8+) as one of the mechanisms of suppression; hence, a granzyme/perforin study of the cell culture is desirable (59, 188).

Second, it is also crucial to confirm cell viability. As Tregs are prone to cell death and can be exposed to prolonged purification processes, it is necessary to confirm their viability throughout the suppression assay (189). To this end, good control should include the presence and absence of IL-2 in the assay.

Finally, in order to obtain correct titers of Treg, they need to be purified. Different purification methods exist, but this is a major challenge given the lack of well-defined markers. Therefore, it is not advisable to purify Treg solely based on the use of CD4- and CD25-labelled magnetic beads. Methods to check for Treg cell purity include 1) phenotypic assessment in resting Treg cells at least 10 days after TCR activation to allow decrease of FoxP3 expression on T cells after activation (190) and 2) evaluation of the TSDR region from the *FOXP3* promoter. Females inactivate one of their X chromosomes by methylation; since *FOXP3* is located on the X chromosome this test may be more feasible in male patients (191, 192), which concstitutes a major limitation in human studies.

Ultimately, since Treg cells exert their effects beyond the classical inhibition of CD4+ T cell proliferation, it would be interesting to also include other Treg-cellular targets, such as antigen-presenting cells (APCs). An interesting proposal would be to measure inhibitory cytokines such as IL-10, IL-35, or TGF-β, although special care must be taken with the available antibody conjugates and with the release kinetics of these cytokines if they are measured with flow cytometry; new strategies to measure cell activation through the ATP/ADP ratio may even be tried. The functional controversies are summarized in **Table 1**.

### 4.3 Concerns regarding Treg cell-based therapies

Treg cell-based therapies present certain concerns that need to be carefully considered. As live cells, Tregs possess the inherent risk of becoming unstable, potentially leading to the generation of undesirable inflammatory responses or exhibiting effector cell properties. Notably, destabilization of FoxP3 and the consequent transformation of Treg into an effector phenotype could trigger tissue destruction. Furthermore, the plasticity of Treg cells allow them to express transcriptional factors that characterize other T cell lineages such as T-bet, GATA-3, and RORγt that can activate transcriptional programs that allow them to migrate and counteract the effect of the cell whose transcriptional factor is matching. This plasticity is a double-edge-sword, as in this context, it makes Treg quite reactive and liable to lose suppressive capacity, secreting inflammatory cytokines under certain circumstances (37, 38). Addressing these issues is crucial to ensure the safety and efficacy of these therapies. The addition of molecules like DNA methyltransferase could help reduce the risk of FoxP3 destabilization. Another factor of concern is the potential risk of oncogene activation subsequent to cell transduction, analogous to occurrences observed in other gene therapy methodologies (37). Moreover, some patients have shown increased susceptibility to opportunistic infections due to the immunosuppressive nature of (38).

To address these issues, strategies to eliminate infused Treg cells if they become overly active or unstable are being thoroughly examined. Among the most advanced approaches are the insertion of suicide casquets or integration sites into the Treg cell, allowing for its inhibition or elimination if necessary (37).

These concerns highlight the importance of ongoing research and vigilance in the development and application of Treg cell-based therapies. Addressing these challenges will be crucial to ensure the safety and effectiveness of these innovative treatments for a wide range of medical conditions. The controversies in Treg cell-based therapies are summarized in **Table 1**.

## 5 Conclusions

The field of Treg has come a long way recently, with its definition and itsfunction-dysfunction studies correlating with pathology and cell-based therapies. Yet some controversies remain. Therefore, continuous update and integration of recent information is needed to offer a response to clinical needs, which arise in patients with PIRD, but also in other immunodeficiencies, allergy, and neoplastic syndromes, in order to develop future therapies aimed at restoring Treg functionality.

## Author contributions

RK-B: Conceptualization, Investigation, Software, Writing – original draft, Writing – review & editing. DA: Conceptualization, Investigation, Software, Writing – original draft, Writing – review & editing. YL: Writing – review & editing. AE-S: Writing – review & editing. AV: Writing – review & editing. RC-R: Funding acquisition, Resources, Supervision, Writing – review & editing. ES-R: Funding acquisition, Project administration, Resources, Supervision, Writing – review & editing. LA: Funding acquisition, Project administration, Resources, Supervision, Writing – review & editing.
